# The Early Diagnosis and Treatment of Insanity
*A postgraduate address delivered in September, 1928.


**Published:** 1928

**Authors:** J. V. Blachford

**Affiliations:** Physician to the Department of Mental Disease, Bristol General Hospital


### THE EARLY DIAGNOSIS AND TREATMENT OF INSANITY.*

BY

J. V. Blachford, M.D.,

*Physician to the Department of Mental Disease, Bristol General Hospital.*

For the purposes of this address I have taken the various types of mental disorder occurring at the different physiological periods, and those connected with the poisons, alcohol, and syphilis, and endeavoured to pick out some points in their incidence which may be of practical value from the practitioner's point of view.

I have here a case of early general paralysis in a woman which illustrates very clearly two facts to which I shall call your attention later on, namely, that any case of acute mania occurring for the first time after the age of 30 should make us consider the possibility of general paralysis ; and that in any case of epileptiform, or transient hemiplegic seizure occurring for the first time about the same age, and with no history of previous fainting attacks, we should look for some organic mischief.

The patient is 33 years of age, married, and has no children. A few months ago she went to the mental hospital, suffering from an attack of sub-acute mania. She was excited, talking nonsense, and thought she was related to the royal family. She soon improved, was discharged on trial, and instructed

* A postgraduate address delivered in September, 1928.

to come to the hospital out-patient mental department. At first she appeared fairly normal, was perhaps somewhat unduly happy, and her aunt who accompanied her stated that at times she made rather foolish and irrelevant remarks. About a fortnight or three weeks after her first visit, as she entered the room she lurched to one side and knocked her head against the door-post ; she was not hurt and laughed about it. Her aunt then told me that since her last visit she had had an attack of partial paralysis in which her right hand and leg had been affected, and that for a time she had almost lost her speech. On questioning her I noticed that this was slightly slow and deliberate, as if she had to think of her words before she spoke. A Wassermann test of her blood showed a triple positive result. She has the Argyll Robertson pupil and her speech is worse than it was a week ago. Evidently hers will be a very rapid case. General paralysis usually commences in the fronto-parietal cortex and invades the basal ganglia later, but in this case they have been involved simultaneously.

Most of you have no doubt come across the child of what may be termed the *young criminal* type. The symptoms occur before puberty, and take various forms. In some the patient appears to tell lies for fun, or because he cannot help it. Sometimes these are romances about themselves, at others false statements about others. In some it takes the form of an almost insane impulse to break things. I know of one small boy who had a present of a fountain-pen, which he suddenly and deliberately broke, although he appeared to value it. The same child would sometimes ask his parents to have a glass on the table moved away from him for fear he would have to smash it. It is a question whether or not some of these are early cases of dementia præcox, but I am inclined to think most are not. They are children of a neurotic temperament, in whom the idea arises and is translated into action before they can consider what the consequences may be. The fact that in the case mentioned the child asked for temptation to be put out of his way shows that on some occasions they can exercise a certain amount of control.

These children should be treated kindly but very firmly. Punishment should not be too severe, but inevitable. Perhaps the best form is the deprivation of pleasures they would otherwise have enjoyed. The cane or birch should be used occasionally, not brutally, but enough to let the recipient realise that it is uncomfortable to be bad.

Where possible the patient should be sent to a boarding-school and the headmaster informed of his condition. If he is kept at home, the relations either forgive his lapses under the impression that they are mental and so unavoidable, or look upon them as the acts of a wicked, selfish boy, which after they have become exasperated they punish unduly.

During puberty and adolescence we have in the first place that form of mental disorder known as *dementia præcox*. Bevan Lewis[1] gives a very good description of this disease in his article on "Adolescent Insanity," but he does not distinguish dementia præcox as a specific disease apart from other forms occurring at this epoch, and consequently gives a very fair recovery rate. If the disorder is one by itself, and most of the recoverable cases merely instances of manic depressive insanity occurring at this time, then dementia præcox appears as a more serious disease, from which there is much less chance of recovery. One is bound to admit, however, that in many of these cases the symptoms are so like those of typical dementia præcox that it is almost impossible to tell the difference, but at the same time they recover. The disease begins between the ages of 15 and 25 ; in a few cases it appears to begin later, but on going into the history carefully there are invariably evidences of eccentricity, etc., earlier in life. There is always a bad family history of either insanity, epilepsy or some neurosis. The patient is generally poor and weedy looking, has a narrow, oval head, the men having too little hair on their faces, the women too much, with a tendency to meeting eyebrows and a moustache. At school they usually avoid the society of their school-fellows, and take very little interest in sport, especially in team games, and if they excel in anything it is self-centred. At the same time, they often are particularly good, in fact almost brilliant, in one particular subject, in women frequently music.

The first evidence of breakdown is an aversion from their relations, generally their parents. Some will turn against one parent in particular, while others appear to lose all interest in and affection for their friends, and do not mind what trouble they give them, nor how much anxiety and worry they cause. They are very apt to adopt mannerisms either of speech, dress, or conduct. Later their loss of interest in their surroundings becomes more marked, they develop a certain degree of negativism, have no initiative and are apt to be impulsive. Still later they develop hallucinations of sight, or hearing, or both, and become deluded. At the same time, however, they are able to recognise their surroundings and to know everything that is going on.

In the early stages, before the disease has become pronounced, in fact when it is suspected but has not been diagnosed, treatment should be stimulative, with attention to the bowels, which are frequently constipated, fresh air, good food, change, and light occupation or employment. Tonics such as nux vomica, and iron in anæmic cases, are often given. When the disease is fully established and the patient certifiable, the best treatment for all concerned is to have him placed in a mental hospital as early as possible. These patients are very impulsive and apt to assault others, especially their parents. They are frequently suicidal. If there is any chance of recovery this is more likely to take place, and that earlier, if they are placed under appropriate discipline and treatment, whereas if the case is hopeless much risk to themselves and others and an enormous amount of worry and anxiety will in this way be avoided.

In cases of dementia præcox the prognosis may be summed up as follows : in all cases it is unfavourable ; in many apparent recovery will take place and the patient may be able to return home for a time, but relapse will occur. In some the patient will become sufficiently demented to be looked after at home. A few will recover. The prognosis is more favourable in females.

The other neurosis which is a frequent cause of insanity during puberty and adolescence is *epilepsy.* This is often undiscovered and unsuspected for a number of years. Sometimes it is purely nocturnal for a long period, in others the attacks are of the petit mal variety, and occurring at long intervals, are overlooked or put down as slight fainting attacks. In investigating a suspicious case the following points are worth considering :—

In the large majority of cases there will be found a history of epilepsy, insanity, or some serious neurosis in the near relatives.

A transient attack of subacute mania passing off in twenty-four to forty-eight hours, so-called mania transitoria, in a patient between 12 and 25 years of age should make us suspicious of epilepsy. It is rare undoubtedly, but does occur occasionally.

A very common history is that of fainting fits, the child having had on more than one occasion to be sent home from school in consequence.

Night troubles, including night terrors, or wetting of the bed occasionally ; occasional morning headaches, with a feeling and appearance of dullness, and tiredness in a child otherwise healthy ; and occasional outbursts of temper out of all proportion to the cause, should suggest a possibility of epilepsy.

Dr. Scripture states that in all epileptics the voice is altered, so that the inflections are blunted, causing it to become wooden. In chronic cases there is no doubt that one can frequently recognise an epileptic by his voice, but in less advanced and milder cases a special apparatus will be needed to discover these changes, of which I have no personal experience.

It was at one time thought that mental trouble was more likely to follow attacks of petit than of grand mal. This appears to be a mistake; it is very seldom one sees a patient with epileptic insanity in an asylum who does not suffer from the major attacks, though it is very common to find from the history of the case that he has at one time suffered from minor attacks.

In the treatment of epilepsy almost every known drug has been tried : silver, until the patient has been literally black in the face, bromides, zinc salts, opium, in fact everything, but during the past few years luminal has undoubtedly taken the first place. It not only reduces the number and severity of the fits, but improves the mental condition, rendering the patient less forgetful, less irritable, and altogether more amenable. Attention to the bowels and general hygiene are of course necessary, otherwise it is not fair to attribute failure to the drug. If you meet with a case in the condition of status epilepticus, a hypodermic injection of a freshly-made solution of luminal soda, 2 grains, may be given in some cases, with really wonderful effect. Luminal itself is only feebly soluble. While upon the subject of luminal soda in a continuous series of fits, I may remark that it also acts beneficially in the convulsive seizures of general paralysis, and Dr. Phillips, of Southmead, tells me he has tried it in three cases of puerperal eclampsia with success.

Migraine is closely related to epilepsy. It occurs in those of a bad family history, commences about puberty, is periodic, and attacks the patient when apparently in good health, and after the attack leaves his health unimpaired. Also patients who suffer from migraine sometimes become epileptics; and luminal, the drug which is most efficacious in epilepsy, appears to be best for migraine.

Of the *insanity of pregnancy* very little experience is gained in mental hospitals. There is naturally a great aversion from putting away a person in that condition, and still more from having the child born in the asylum.

The symptoms are generally those of despondency, the patient, even if mildly maniacal, having a fear that something dreadful is going to happen. If possible the patient should be nursed at home and watched carefully in case of suicidal attempts. As soon as the child is born it should be taken away, and then if the mother becomes worse the advisability of placing her in a mental institution should be considered.

In *puerperal insanity* it is well to give a brisk purge; in some cases the patient will improve from that time. It is surprising what a number of cases are sent in to mental hospitals not only from private houses, but from public institutions, in whom the bowels are loaded and the bladder full. I have known cases in which the latter was so pronounced that the matron has passed a catheter before having the patient washed, for fear of an accident.

If in spite of an aperient and appropriate treatment the patient does not show marked improvement within a day or two, the friends should be persuaded to have her placed in a mental hospital without delay. The difference in the recovery rate in those put away early and those in which this proceeding has been delayed is so marked as to be almost incredible. You may take it as a rule that if the patient is under 30 and is put away within a week, the prognosis is very favourable and her residence in the asylum will not be long. For every day's delay the prognosis becomes less favourable both as regards ultimate recovery and the time that will elapse before it takes place. The recovery rate for all cases admitted is about 80 per cent., but Bevan Lewis[2] states that in 24 consecutive cases admitted to the West Riding Asylum under 30 years of age, and within a week of confinement, 13 cases recovered within three months, and the remaining 11 within five months, and that in spite of a bad family history in several of the cases. This is perhaps exceptional, but illustrates the necessity for early treatment.

In the great majority of cases occurring at the *change of life* the symptoms are those of self-accusatory depression. The patient thinks she has not been a good mother or wife, and that any trouble in her family is due to her carelessness or neglect. If the husband goes out shopping the patient fears he has met with an accident, and thinks she ought to have accompanied him. The prognosis is good if the patient is placed under treatment early, and cases of climacteric insanity once having recovered seldom recur, but early treatment is essential. In this connection I well remember a particularly sad case.

A woman in good circumstances broke down at the climacteric, became depressed and at times maniacal. Her father had died in an asylum, her husband was dead, and she had such a dread of institutions that she had extracted from her only child a promise that if she ever became "queer in her head" she would not have her put away. Her daughter was advised by two specialists to have her sent to a private mental hospital or nursing home, but she refused. When I saw her she was lying in bed in the condition of a person in the last stage of drunkenness, could not appreciate her surroundings, would look up if spoken to sharply, and was passing everything under her. On making enquiry it was found that she was not taking much stimulant, but had been having 15 grains of sulphonal every day for six weeks with occasional doses of bromidia at night time. The sulphonal had been ordered by one of the consultants, but he had no idea that it would be continued indefinitely. The medical man in charge said he had had no experience of such cases. The daughter was told that her mother was in a very serious condition, but that if she could survive the next few days she would have a good chance of recovery if she were placed in a proper mental hospital. Unfortunately, persuasion was unavailing, and the patient was nursed at home. She recovered her bodily health, but her mental condition only improved up to a certain point, and this took from eighteen months to two years to come about. Now she is childish and moderately demented, and though she can mix with her friends at times, has not that keen appreciation of the pleasures of life which she had formerly, and which she would have had if appropriate treatment had been adopted.

Climacteric insanity may arise a considerable time after the menopause. It is often complicated by alcohol, but in these cases the habit is the consequence and not the cause of the mental condition.

*Senile* patients are always a cause of great anxiety. If their means are sufficient and they are not too noisy, they can frequently be looked after at home. They require constant supervision to prevent their falling about, and are apt to be very sleepless and noisy, especially at night. A very good and safe drug for this condition is dial, a grain and a half; or paraldehyde, two to three drachms, if the patient can be persuaded to take it. Two drachms of bromidia at night is also often useful, but should not be given to keep the patient quiet during the day. If these patients refuse food, they should of course be certified at once. As to poorer patients, the sooner they are sent to a mental hospital the better.

The early symptoms of *delirium tremens* are no doubt well known to you all, but opinions differ as to the line of treatment to be adopted. In the first place it is not wise to stop the administration of all alcohol at once, especially in recurrent cases in which the heart muscle is usually degenerate. Where all alcohol is suddenly stopped the risk of sudden collapse and death is considerable. In some institutions the routine treatment for all these cases was to give a brisk purge, knock off alcohol and give plenty of water, and in one of these it was not at all uncommon for the medical officer to be summoned by the attendant in charge to a newly-admitted case, asking him to come at once as the patient had had a seizure. The patient was usually dead when he arrived of heart failure, due to the sudden cessation of all stimulants. The amount of stimulant should undoubtedly be considerably reduced, and after the first twenty-four or forty-eight hours all spirits be discarded, but even then it is as well to give beer or stout with meals, and as soon as the patient can eat and sleep well all alcohol should be stopped.

It is in delirium tremens that chloral hydrate finds its chief rôle, not in continuous doses but one large one, after which the patient will frequently have a prolonged sleep and wake up a different man.

There is just one point to mention in connection with this subject. The term dipsomaniac is frequently misapplied. The ordinary drunkard who drinks through habit, sometimes acquired through bad company, sometimes through trouble, is not a dipsomaniac, and if by any means he can be persuaded to give it up may be reclaimed, but not so the dipsomaniac. The latter may give up drinking and "go on the tea-pot," as he calls it, for weeks at a time, then suddenly has the craving, and though he fights against it, almost invariably succumbs, often feeling very much ashamed of himself all the time. It is a form of impulsive insanity, and is, I believe, incurable. It is only less desperate than morphinomania.

Forms of mental disorder caused by *syphilis* may be divided into two groups, general paralysis and cerebral syphilis. General paralysis is essentially a disease of the nerve cells. Cerebral syphilis affects the cells secondarily, probably through interference with their blood supply.

The earliest stages of general paralysis are very insidious in their onset, and are generally marked by mental symptoms only, though in certain cases the first noticeable symptom may be a seizure. Apart from this the early signs may be summarised as follows :—

1. First attack of subacute mania between 30 and 50.

2. Lapses of memory, the patient sometimes forgetting a word in the midst of a sentence. At other times he will go on an errand and quite forget his mission.

3. Exaggeration of natural characteristics. General paralysis has very much the same effect as alcohol. At first the last-established centres of control are paralysed. Those latest controlling centres which restrain him in so many of his words and actions are in abeyance and the man has become " himself." I remember a striking instance of this.

A few years ago a woman whose husband I had been asked to examine wrote saying that she thought she ought to let me know his symptoms as far as possible. Amongst other things she said, " My husband has always been a selfish man, but lately his selfishness has become almost unbearable." That struck me as being a very suspicious symptom. On examination the physical signs were almost absent, but he had lapses of memory, could not concentrate as he had been wont to, felt his customary work too much for him, and that there was something wrong in his head. On being questioned he admitted having contracted syphilis seventeen years before. He proved to be a general paralytic and went down hill rapidly.

4. Undue energy.

5. Inability to concentrate as formerly.

6. Rashness in business.

Later on the pupils, tongue and speech become affected, but it is important to diagnose the case in the early stages in order to guard the patient and his family from his early moral lapses and want of control.

The Wassermann test is perhaps the most accurate and convincing when it can be obtained, but though a positive blood test combined with undoubted symptoms of general paralysis may be considered conclusive, unless the clinical symptoms are very definite one must have the cerebro-spinal fluid tested. There are many patients with a positive blood test who are general paralytics, and there are others suffering from cerebral syphilis, not general paralytics, in whom the blood test is positive but the cerebro-spinal fluid negative. This is probably why, in some cases, treatment with iodide causes the symptoms to remit and the patient to improve considerably. I have seen several cases of general paralysis in which there was such a marked change that it was hoped a mistake had been made in the diagnosis, but after a short time they broke down and went the way of all general paralytics. This may be due to the fact that many general paralytics have cerebral syphilis as well, and that the symptoms are due to disease of the vessels interfering with the blood supply. This condition is improved by the anti-syphilitic treatment, and if the general paralysis is in its early stages the improvement is such as to deceive us and make us hope for complete recovery, but if general paralysis is really established, as far as our present experience goes, the case may be considered hopeless.

The early treatment of general paralysis is most important. In any suspected case wherever possible the patient should be prevented from transacting any business, handling any money or being able to sign cheques. As Savage used to say, "Take away his cheque-book and send him away from his business." If he can be sent for a voyage, or for a prolonged stay in the country with a friend, time will be gained for confirming the diagnosis. If he is not a general paralytic no harm will have been done. If he is, his estate may have been saved from ruin.

The medical treatment of these cases is very unsatisfactory ; antisyphilitic treatment sometimes brings about temporary improvement, only to give rise to disappointment. All kinds of treatment by intrathecal and intraventricular injections have been tried without success. Of the latest, by malaria infection, I cannot speak from personal experience. The mortality rate is high, and it is only likely to be beneficial in early cases, so early that the diagnosis in many appears to be uncertain.

*Cerebral syphilis* differs from general paralysis in being amenable to antisyphilitic remedies. It may affect any part of the cerebrum, but for purposes of description may be divided roughly into :—

1. Cases simulating general paralysis.
2. Those suffering from hemiplegia, with or without loss of speech.
3. Cases of rapidly oncoming amaurosis with dementia.

Instead of discussing these at length, it will be better to quote three typical cases :—

A man found unconscious in the street was admitted into the Bristol General Hospital. He spoke very little, the pupils were enlarged and fixed to light, he was morose, took no interest in anything, and at times struck at the nurse who took him his meals under the delusion that she was his wife. His symptoms were very like those of a person suffering from fairly advanced general paralysis, but from his mental state he was considered to be the subject of cerebral syphilis. He was too troublesome to be kept in the hospital, and so was sent to an asylum. On making further enquiry it was discovered that seven years previously he had been in another asylum and considered a general paralytic. The Wassermann test for his blood was triple positive, but that for his cerebro-spinal fluid negative. He was given potassium iodide at first, 5 grains t.d.s., increased rapidly to 30 grains t.d.s. In a few weeks he was working about in the ward, quite sensible and good tempered, and I believe has since been discharged.

A young woman, about 26, was admitted to the General Hospital with right hemiplegia. She either could not or would not speak, was strange in her manner and depressed. Her condition was thought to be due to cerebral syphilis, probably a localised meningitis. Her blood was triple positive and her cerebro-spinal fluid negative to the Wassermann test. She was treated with iodide, soon recovered her speech and the use of her right side, and was sent to the Convalescent Home for a fortnight. Either while there or shortly after she had a sudden attack of cerebral hemorrhage, with left-sided hemiplegia and died. Post mortem, on the left side was a fair amount of basal meningitis, evidently clearing up, which had been the cause of her first attack. On the right side a small aneurism had formed in one of her cerebral vessels and had burst.

Of the third group a case was admitted to the Bristol Asylum many years ago. There was a history of syphilis. In a few months from the commencement of his mental trouble he had become almost blind and quite demented. On post-mortem examination his anterior corpora quadrigemina and optic thalami were found markedly degenerate.

Some time afterward, about the year 1900, two cases were admitted with symptoms similar to those of the last case. Both had a history of syphilis, and in both the illness which had necessitated their being put away commenced a few months before, with rapidly increasing loss of sight and dementia. On admission the dementia was most profound, more than in a case of general paralysis or even a congenital epileptic. Neither could take interest in anything, they could not stand up, but lay on the floor of a single room and passed everything under them. They were treated with iodide, and in both there was considerable improvement in a short time. In from three to four months they were working about in the ward and taking a fair interest in life. In one the sight had much improved, in the other had become apparently normal. Unfortunately both had to be transferred to another asylum and so were lost to sight, but twenty years after the Gloucester police rang up to make some enquiries about one of them who had just got into their hands, so that he must have been outside an institution and still alive. These cases are of extreme interest and importance, because if diagnosed sufficiently early they can be cured. Otherwise the brain becomes irretrievably damaged, and if no treatment is undertaken they gradually sink and die.

Generally speaking, in the early stages of most forms of mental disorder the treatment should be stimulative. In both melancholia and mania the nerve cells are run down and require feeding and not depressing, so that in most cases bromide should be avoided and chloral hydrate never given, the patient encouraged to take as much food as possible, with rest, fresh air and attention to bowels.

In very acute cases, when it is imperative to quieten the patient, perhaps the best treatment is a hypodermic injection of hyoscine hydrobromide gr. 1/50 and morphia gr. $\frac{1}{4}$, then before the patient has recovered sufficiently to be too resistive it may be possible to give some other sedative, as paraldehyde, sulphonal, or veronal.

Hyoscine ought not to be given either hypodermically or by the mouth, except in very acute cases, and even then only once or twice. It dries the throat, paralyses accommodation and makes the patient think he is being poisoned, which as a matter of fact is the case. It will cause him to refuse his food, and if it is continued for long he will develop hallucinations and behave much like a patient suffering from delirium tremens.

Chloral hydrate is a very dangerous drug, especially in those with any heart trouble. It will, however, be found beneficial in delirium tremens, where it is almost a specific. In sleeplessness in acute cases it may be frequently given with advantage, preferably in the form of bromidia, one, two or even three drachms at night time. In acute epileptic conditions it may be used, but here luminal soda may well be tried first. Chloral should never be given continuously to keep a patient quiet, and when for any reason it is administered great care must be taken to keep him warm, otherwise there is grave danger of hypostatic pneumonia.

Paraldehyde is one of the safest hypnotics we possess. The only objections are its odour and taste; but patients soon become accustomed to these, and if the room be airy and well ventilated the smell will not be particularly disagreeable. Any amount up to 4 drachms may be given with safety. I have known patients who have become quite fond of it, in fact almost developed a paraldehyde habit, and after all it is very much the same as taking a "night-cap" of whisky.

Sulphonal is frequently of great value. It is best to give a good dose, 50 to 60 grains; then if the patient is restless the next day to give 30 to 40 grains, then to substitute something else. When sulphonal is being taken careful attention must be paid to the bowels. A reliable preparation is essential. Veronal, which is synonymous with diethyl-barbituric acid and barbitone, is also at times useful in acute cases given in 30-grain doses, but like sulphonal should not be given continuously. Medinal is the soda salt of veronal, and may be given in from 30 to 40 grain doses. It is very soluble in water, and not disagreeable to the taste.

Dial ciba, generally called dial, is useful in cases of sleeplessness in 1½ grain doses, and in somewhat larger doses may be useful in more acute cases.

In some cases of irritable, restless melancholia tincture of opium is a valuable and safe drug, especially combined with spt. ether. sulph. The dose of opium may be increased from m 15 up to m 30 t.d.s., and in these cases frequently does not appear to affect the bowels. In maniacal cases it usually does more harm than good.

As regards the other forms of treatment of early cases some advocate prolonged rest in bed and isolation. In patients who are emaciated and exhausted it is well to adopt it, but one can hardly imagine anything more at variance with common sense, or likely to increase the patient's delusions, than to shut up in a room by himself one who is depressed, who thinks he has committed the unpardonable sin and is to be damned for ever, and can think of nothing but himself, without anything to distract his attention, and allow him to go on all day long worrying over his own condition. I have come across patients who have been through this treatment, and well remember one who had been in a well-known endowed institution, where the medical officer insisted on a month's rest in bed as preliminary treatment; and although he had nothing to complain of the food or other treatment, hated every minute of the time and the place, but was loud in his praises of the London County Asylum to which he was subsequently transferred, in which enforced rest was not part of the treatment.

Of the so-called treatment by psycho-analysis I can find nothing in its favour. When you have to make your diagnosis by means of dreams and odd associations, it seems too much like the old metaphysics. Commercially it is excellent, scientifically it is worse than useless, professionally the less said about it the better.

In most cases the question of certification arises sooner or later, and a decision has to be taken as to what is best for the patient and his friends.

A patient is legally certifiable when on account of his mental state he is dangerous to himself or others, or offensive to the public decency.

If a person though irresponsible and faulty in habits is old, infirm and bed-ridden, he should be nursed at home, if his friends can afford to pay for proper nursing and medical attendance. Otherwise certification is the only alternative in the interests of the patient, and in those cases it is best to send to the relieving officer of the district and leave him to do the rest.

In other cases it may be quite different. If the patient is actually dangerous, you will as a rule have little trouble in persuading his friends to have him put away; but if he does not appear to them to be so, though in your estimation he is likely to become so, as in many cases of dementia præcox, it is often advisable to suggest that he place himself in a private mental hospital as a voluntary boarder. The patient will often consent to this without much demur, and the friends will fall in with the suggestion when they realise that this can be done without certification. If after a time the patient becomes too troublesome or, though not considered fit to be at large, gives notice that he wishes to leave, the proprietor of the mental home will inform the friends, and will tell them either to come over and petition for his detention under certificates or remove him. As a rule the relief which they have experienced by his removal from home, after the turmoil and anxiety they have recently experienced, has been so great that they will go over at once and not hesitate to have him certified.

To be a voluntary boarder the patient must know where he is and what he is doing.

If a patient obstinately refuses all food he ought to be certified as soon as possible, otherwise valuable time will be lost, and every day's delay will lessen his prospects of recovery.

In these cases we cannot feed forcibly, that is with a tube, except in those who are under age, and then only with the consent of the parents. Even in the case of a voluntary boarder forcible feeding might be considered an assault. The proprietor of a private asylum once asked a legal commissioner if he would be safe in feeding a voluntary boarder forcibly. His reply was, "Yes, provided you have the patient certified next day."

REFERENCE.

[1] *Text-Book of Mental Diseases*, p. 370.

